# Non-canonical Wnt/Ca2+ signaling is essential to promote self-renewal and proliferation in colon cancer stem cells

**DOI:** 10.3389/fonc.2023.1121787

**Published:** 2023-03-10

**Authors:** Miguel Angel Sarabia-Sánchez, Angela Patricia Moreno-Londoño, María Cristina Castañeda-Patlán, Eduardo Alvarado-Ortiz, Juan Carlos Martínez-Morales, Martha Robles-Flores

**Affiliations:** Departamento de Bioquímica, Facultad de Medicina, Universidad Nacional Autónoma de México (UNAM), Mexico City, Mexico

**Keywords:** cancer stem cell, non-canonical Wnt/Ca2+ signaling, PLC, NFAT, tumor spheres

## Abstract

**Introduction:**

Cancer Stem Cells (CSC) are responsible for maintaining tumor growth, chemoresistance, and metastasis. Therefore, understanding their characteristics is critical to progress in cancer therapy. While the contribution of the canonical Wnt/b-catenin signaling in both normal and CSCs had been well established, the function of non-canonical Wnt signaling cascades in stem cells is unclear. Recently, we reported that Wnt ligands trigger complex signaling in which the canonical and non-canonical responses can be simultaneously activated by one ligand in colon cancer cells, suggesting, therefore, that noncanonical Wnt pathways may also be important in CSCs.

**Methods:**

The present work aimed to know the role of the Wnt/Ca2+ pathway in colon CSCs. We used tumorspheres as a model of CSCs enrichment of CRC cell lines with different Wnt/b-catenin contexts.

**Results:**

Using Wnt3a and Wnt5a as prototype ligands to activate the canonical or the non-canonical pathways, respectively, we found that both Wnt3a and Wnt5a promote sphere-formation capacity and proliferation without stimulating b-catenin-dependent transcription. Upregulation of sphere formation by Wnt5a or Wnt3a requires the downstream activation of Phospholipase C and transcriptional factor NFAT. Moreover, the single specific inhibition of PLC or NFAT, using U73122 and 11R-VIVIT, respectively, leads to impaired sphere formation.

**Discussion:**

Our results indicate that both types of ligands activate the Wnt/Ca2+ signaling axis to induce/maintain the self-renewal efficiency of CSCs, demonstrating to be essential for the functions of CSC in colon cancer.

## 1 Introduction

Stem cells are a population of cells characterized by the capacity for cell self-renewal and for giving rise to mature cells in a given tissue. Their regulation is essential to support embryo development, organogenesis, and homeostasis of tissues in the body since they are required for tissue replacement throughout the human lifespan (1). The disruption of stem cell functions leads to several human diseases, such as cancer. Cancer stem cells (CSCs) represent a cell subset with characteristics akin to healthy stem cells, like self-renewal capacity, but with tumor-initiating ability and high invasive capacity. These properties of CSCs are thus involved in tumor initiation, propagation, and relapse.

Canonical Wnt signaling has been defined as one of the most important regulators of normal and cancer stem cells (2–4). While it is well established that Wnt/β-catenin signaling is aberrantly activated in most colorectal cancers (CRC), the implication of the non-canonical, non- dependent β-catenin Wnt signaling in carcinogenesis and CRC progression is still unclear (4-7). With respect to the role played by Wnt signaling in stem cell functions, it has been established that the canonical Wnt/β-catenin signaling cascade is involved in the self-renewal of stem cells and proliferation or differentiation of progenitor cells (5–7). However, although it is well known that non-canonical Wnts regulate cellular polarization, control nuclear localization of NFAT transcriptional factor, suppresses canonical Wnt signaling, and promote invasion, survival, and metastasis of CSCs, the contribution of non-canonical cascades to stem cells biology is still not well defined.

We have recently reported that both canonical prototype Wnt3a ligand and non-canonical prototype Wnt5a ligand activate a typical Wnt/Ca2+ non-canonical signaling pathway in malignant colon cells, promoting Ca2+ mobilization as a result of phospholipase C (PLC) activation and inducing cell migration in these cells (8). Importantly, we demonstrated that canonical Wnt3a ligand not only stimulated the β-catenin transcriptional activity in colon cancer cells but, at the same time, activated PLC, promoted Ca2+ mobilization, and induced Rho kinase and PLC-dependent cell migration (8). These results, therefore, indicated that Wnts might simultaneously activate canonical and non-canonical Wnt signaling in colon cancer cells (8).

The aim of this work was to examine the role played by the non-canonical Wnt/Ca^2+^pathway, specifically in colon cancer stem cells. Using the prototype non-canonical ligand, Wnt5a, in comparison with Wnt3a, the prototype of a canonical β-catenin activating ligand, we describe here that non-canonical Wnt/Ca^2+^ cascade plays an essential role in inducing and maintaining the self-renewal capacity of colon cancer stem cells.

## 2 Materials and methods

### 2.1 Cell lines

All cancer cell lines and the L-Wnt3a or L-Wnt5a cell lines used here were purchased from American Type Culture Collection (ATCC; Manassas, VA, USA). RKO is a prototype of BRAF-driven colon cancer cells and exhibits responsive canonical Wnt signaling. SW480 and SW620 are the prototype of KRAS-driven colon cancer cells and have a truncated version of APC. SW480 and SW620 were obtained from the same patient from the primary tumor and lymph node metastasis, respectively.

### 2.2 Monolayer culture

RKO was maintained in Dulbecco’s modified Eagle’s medium (DMEM) supplemented with 10% Fetal Bovine Serum (FBS), 2 mM L-glutamine, and antibiotics (100 U/ml Penicillin, 100 µg/ml Streptomycin and 25 µg/ml amphotericin). SW480 and SW620 were maintained in DMEM F-12 supplemented with 5% FBS, 2 mM L-glutamine, and antibiotics (100 U/ml Penicillin, 100 µg/ml Streptomycin and 25 µg/ml amphotericin). L-Wnt3a cells were maintained in DMEM supplemented with 10% FBS and L-Wnt5a cells were maintained in DMEM supplemented with 10% FBS and 4 mM L-glutamine. All cell lines were cultured at 37°C in an atmosphere of 5% CO_2_.

### 2.3 Sphere culture

Cells were collected from monolayer standard culture by trypsinization. Viable cells were quantified by staining with Trypan blue and were seeded to 1 cell/µl in serum-free medium containing DMEM F-12 supplemented with 1X B27, 20ng/ml of EGF and 1X antibiotics (100 U/ml of Penicilin, 100 µg/ml of Streptomycin and 10 µg/ml of Gentamicin and 25 µg/ml Amphotericin) in ultralow-attachment plates. Fresh medium was added each third day. For further analysis or sequential cultures, spheres were dissociated into single cells using TrypLETM Express (Gibco Cat no. 12604-013) according to manufacturer instructions. Sphere forming efficiency (SFE) was evaluated using the formula: [number of spheres/number of seeded cells] x 100.

### 2.4 Plasmids

The plasmids used in this work were the following TOP-GFP-mCherry plasmid was a gift from Ramesh Shivdasani (Addgene #35491). M50 Super 8x TOPFlash (Addgene #12456) and M51 Super 8x FOPFlash (Addgene #12457) were a gift from Randall Moon. pCMV/mCherry, pSORE6/mCherry, pCMV/GFP, and pSORE6/GFP (which contains a tandem repeat of the response element to Oct4/Sox2 derived from Nanog promoter) were a gift from Marco Velasco Velázquez.

### 2.5 Wnt ligand treatment

The cells were stimulated in the absence or the presence of recombinant human Wnt-3a (Cat. No. 5036-WN-010 R&D Systems) or Wnt-5a (Cat. No. 645-WN-010 R&D Systems). When indicated, the conditioned medium, obtained from L-Wnt3a or L-Wnt5a cell lines, was used according to the manufacturer’s instructions.

### 2.6 TOP reporter gene assay

The β-catenin-dependent transcription was evaluated using the plasmid TOP-GFP-Mcherry. Briefly, the cells were lentivirally transduced with TOP-GFP-mCherry. Transduced cells were isolated using FACSaria sorter to isolate mCherry^+^ cells. Positive fluorescence to mCherry^+^ was corroborated by cytometry flow using Attune Nxt cytometer. Positive cells were seeded in sphere culture and stimulated each third day with DMSO (vehicle), 250 ng/ml of Wnt3a, or 250 ng/ml of Wnt5a. The 11^th^ day of sphere formation, spheres were disaggregated at cell single and analyzed to detect GFP^+^ cells, β-catenin-dependent reporter, by cytometry flow using Attune Nxt cytometer.

### 2.7 Lentivirus generation and cell transduction

Lentivirus was generated by cotransfection with the plasmids pCMV-VSV-G (Addgene #8454) and pCMV-dR8.2 (Addgene #8455) together with the plasmid of interest in HEK-293Tcells. Lentiviral particles were harvested 72 hours post-transfection, aliquoted, and frozen at -80°C until use. For transduction with lentiviral constructs, target cells were exposed to viral supernatants for 24 h with Polybrene (5-20 µg/ml). Transduced cultures were selected with 5-10 µg/ml of Puromycin for ten days in the case of plasmids that had resistance. For plasmids that do not allow selection for antibiotic resistance, lentiviral transduced cells were cell-single sorted.

### 2.8 Intracellular calcium determinations

The calcium was quantified as previously described (8). In brief, the cells were serum-starved for 1 h at 37°C. Then, cells were loaded with 2.5 µM Fura-2/AM (Cat. No. F1201 Invitrogen) in Krebs-Ringer-HEPES containing 0.05% BSA, pH 7.4, for 1 h at 37°C. The cells were washed and incubated 5 min in the absence or presence of 4 μM PLC inhibitor U-73122. Fluorescence measurements were carried out with Wnt3a or Wnt5a ligands at 340- and 380-nm excitation wavelengths and at 510-nm emission wavelength with a chopper interval set at 0.5 s, using an AMINCO-Bowman Series 2 luminescence spectrometer (Rochester, NY).

### 2.9 Pharmacological treatment

U73122, obtained from Sigma-Aldrich (St Louis, Mo, USA), 11R-VIVIT (Merck Cat. no. 480401), or vehicle (DMSO), were added at indicated concentrations during spheres formation each third day. Regarding the specificity of these compounds: U73122 is a potent, selective, and cell-permeable inhibitor of C-type phosphatidylinositol-specific phospholipases that inhibits agonist-induced phospholipase C activation (IC50 = 1-2.1 μM), but has also reported inhibiting 5-lipoxygenase. 11R-VIVIT is a competitive NFAT inhibitor peptide based on the conserved calcineurin docking site of the NFAT family. Referred to as VIVIT, this peptide interferes with calcineurin-NFAT interaction without disrupting calcineurin phosphatase activity and without affecting other signalings regulated by calcineurin, such as NFKB (9).

### 2.10 Cell viability

Spheres were treated with U73122 or 11R-VIVIT each third day at the indicated concentrations. Quantification of viable cells was carried out on the 11^th^ day of spheres forming using Propidium Iodide at 1:2000 dilution, and fluorescence was measured in the ATTUNE NEXT equipment.

### 2.11 Cellular fractionation

Separation of cytoplasmic and nuclear proteins was performed as briefly described below. Spheres were centrifuged at 2,800 rpm for 9 min. Pellet was resuspended in cytoplasm homogenization buffer I (10 mM HEPES, 1.5 mM MgCl_2_, 10 mM KCl, 0.5% Triton X-100, pH8) and shaken vigorously. Lysates were incubated at 4°C for 15 min, and 1% NP-40 was added and shaken vigorously. Lysates were centrifuged at 900 rpm for 10 min at 4°C. The supernatant was recovered and stored as a cytoplasmic extract. The pellet was resuspended in nucleus homogenization buffer (5 mM HEPES, 0.75 mM MgCl_2_, 5 mM KCl, 0.25% Triton X-100, 0.5 M NaCl, 0.1 M EDTA, 10% Glycerol, 0.25 mM DTT) and shaken vigorously. Lysates were incubated at 4°C for 15 min and centrifuged at 13,000 rpm for 5 min. The supernatant was recovered and stored as a nuclear extract.

### 2.12 Western blotting

Cells were lysed with RIPA buffer (50 mM Tris-HCl, pH 7.4, 150 mM NaCl, 1 mM EDTA, 0.5% sodium deoxycholate, 1% NP-40, 0.1% SDS, and supplemented with protease and phosphatase inhibitors) and shaken vigorously every 10 min for 3 h. Lysates were centrifugated (13000 rpm for 5 min), and supernatants were stored at −80°C until use. Proteins were quantified by Bradford protein assay and separated by SDS-PolyAcrylamide Gel Electrophoresis (SDS-PAGE). The proteins were transferred to nitrocellulose membranes (Bio-Rad), and membranes were blocked with 3% nonfat dry milk in TBS for 1 h. Primary antibodies incubated overnight at 4°C with the indicated primary antibody (Non-phospho (Active) β-catenin (Cat. No. 8814 Cell Signaling), NFAT1 (Cat. No. 610703 BD Biosciences), NFAT2 (Cat. No. 556602 BD Biosciences), NFAT3 (sc-271597 Santa Cruz Biotechnology), NFAT4 (SC-8405 Santa Cruz Biotechnology), Lamin B1 (ab16048 Abcam), α-Tubulin (T9026 Sigma)] according to manufacturer’s instructions. Then, membranes were washed with washing buffer (TBS, 0.025% Tween 20) and incubated with HRP-conjugated secondary antibody for 2 h at room temperature. Blots were revealed using SuperSignal Kit (Pierce) in C-DiGit Blot scanner (LI-COR Bioscience, Lincoln, NE, USA), and analysis was performed by Image Studio™ Lite Software (LI-COR Biosciences).

### 2.13 Flow cytometry

Cells were obtained by trypsinization or using TrypLETM Express from monolayer or sphere culture. The cells were incubated for 30 min at 4°C as follows: CD133-PE-coupled (Cat. No. 12-1338-42 eBioscience), CD44-APC-coupled (559942 BD Pharmingen), and CD44v6 (Cat. No. BMS125 eBioscience). In the case of CD44v6, the cells were washed and incubated with FITC-coupled anti-mouse secondary antibody for 30 min at 4°C in dark conditions. The Single cell suspension was acquired in Attune Nxt cytometer. Data were analyzed using the FlowJo software (Tree Star^®^). For FACS of cells transduced with the SORE reporter system, RKO^CMV^ and SW480^CMV^ were used as control of fluorescence, and cell sorting was performed using a FACSaria sorter.

### 2.14 *In silico* analysis

Gene signature quantification was performed using Gene Expression Profiling Interactive Analysis (GEPIA2) platform considering the median group cutoff (10). For analysis of CAMK2B mRNA in different stages of American Joint Committee on Cancer, TCGA public data from Pan- Cancer was used (11).

### 2.15 Statistical analysis

The data is represented as the mean ± Standard Error of the Means (SEM) of at least three independent experiments. Student’s t-test was used to compare the means of two groups. Multiple comparisons between three or more groups were performed using one-way analysis of variance (ANOVA) followed by Bonferroni’s multiple comparisons. A p-value of p<0.05 was considered statistically significant.

## 3 Results

### 3.1 Tumor sphere culture is enriched in CSCs and serves as a study model of CSCs derived from colon cancer cell lines

**Figure 1:**
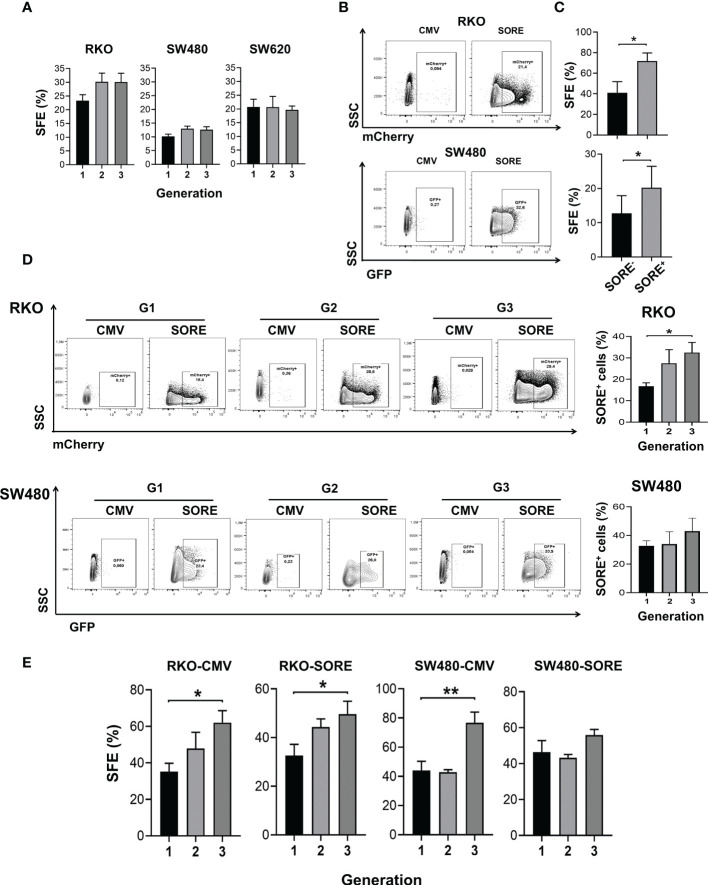
CSC are identified in spheres cultures derived from colon cancer cell lines. **(A)** Quantification of SFE of RKO, SW480, and SW620 cells throughout three consecutive generations. **(B)** Density plots of RKO and SW480 cells transduced with pSORE6/mCherry or pSORE6/GFP, respectively, and measured by flow cytometry. Transduced cells with pCMV were used as a reference for fluorescence. **(C)** Quantification of SFE of single cell cultures sorted from SORE6^-^ and SORE6^+^ subpopulations of RKO^SORE6^ and SW480^SORE6^ populations. **(D)** Density plots of RKO^SORE6^ and SW480^SORE6^ throughout three consecutive generations (left panel). Quantification of the percentage of SORE6^+^ subpopulation of each generation (right panel). **(E)** Quantification of SFE of RKO^CMV^, RKO^SORE6^, SW480^CMV^, and SW480^SORE6^ throughout three consecutive generations. Data are represented as the mean values ± SEM of at least three independent experiments using Student´s t-test. **(A, D, E)** or one-way ANOVA followed by Bonferroni´s multiple comparisons. *p<0.05; **p<0.005.

Colon spheres have been widely described as a stable *in vitro* model to study stem cells since their enrichment has been demonstrated in these cultures (12). To establish whether RKO, SW480 and SW620 colon cancer cell lines have the capacity to grow in non-adherent conditions, cultures in a clonal density upon anchorage-independent growth and serum-free medium were performed, and the sphere formation efficiency (SFE) was quantified. In each cell line, a cellular subpopulation survived and generated spheres. In particular, SW480 had the lowest SFE, while RKO and SW620 had similar SFE (**Figure 1A**). The self-renewal capacity is recognized through consecutive cultures, where spheres from the first generation are dissociated at cell single and seeded to form spheres in a subsequent generation and so on. As can be observed in **Figure 1A**, the quantification of SFE in the first, second and third generations showed that RKO, SW480 and SW620 cells had a cellular subpopulation able to maintain the capacity to form spheres (**Figure 1A**). This supported the notion of the self-renewal process and then that the spheres are constituted of stem cells.

The expression of several stem cell markers, such as CD133, CD44, and CD44v6 has been widely validated in colon CSCs (13, 14). Consistent with this, the analysis of cell surface expression of these markers by flow cytometry in second-generation spheres showed that more than 95% of RKO or SW480 spheres express CD44 and display a heterogeneous expression of CD133 and CD44v6 depending on the CRC cell line spheres tested (**Supplementary Figure 1**). Transcriptional factors OCT4, SOX2, and NANOG have also been reported to be relevant for stemness (15). Thereby, a reporter plasmid system referred to as pSORE6, containing a tandem repeat of an OCT4/SOX2 response element derived from the NANOG promoter, was used to confirm the presence of CSCs in the spheres. Analysis by flow cytometry detected SORE6+ cells in RKO and SW480 spheres transduced with pSORE6 (RKO^SORE6^ and SW480^SORE6^, respectively) compared to cells transduced with pCMV, the control plasmid lacking SORE element (RKO^CMV^ and SW480^CMV^) (**Figure 1B**). To corroborate that SORE6+ cells are enriched in CSC, SFE of single cell culture from SORE6+ cells and SORE6- cells was assessed. Results showed a higher SFE in SORE6+ cells compared to SORE6- cells, both in RKO^SORE6^ and SW480^SORE6^, reinforcing the notion that SORE6+ cells have a higher capacity to form spheres than SORE6- cells (**Figure 1C**). Once SORE6+ cells were identified in RKO^SORE6^ and SW480^SORE6^ populations, it was of interest to know whether this subpopulation is maintained throughout consecutive sphere cultures. The analysis showed that the percentage of SORE6+ cells was also increased in the third generation but only statistically significant in RKO^SORE6^ (**Figure 1D**). Because the percentage of SORE6+ cells was augmented in subsequent sphere culture, SFE was measured in RKO^SORE6^ and SW480^SORE6^ populations to find out if it was related to an increase in the proportion of cells able to form spheres. Results showed that SFE improved in the third generation of RKO^SORE6^ spheres, while SW480^SORE6^ exhibited similar percentages (**Figure 1E**). Additionally, RKO^CMV^ and SW480^CMV^ had enrichment of sphere-forming cells, corroborating that the sphere culture is a tool to enrich the CSC subpopulation (**Figure 1E**).

### 3.2 Wnt3a but not Wnt5a stimulates β-catenin transcriptional activity in RKO and SW620 sphere cells

**Figure 2:**
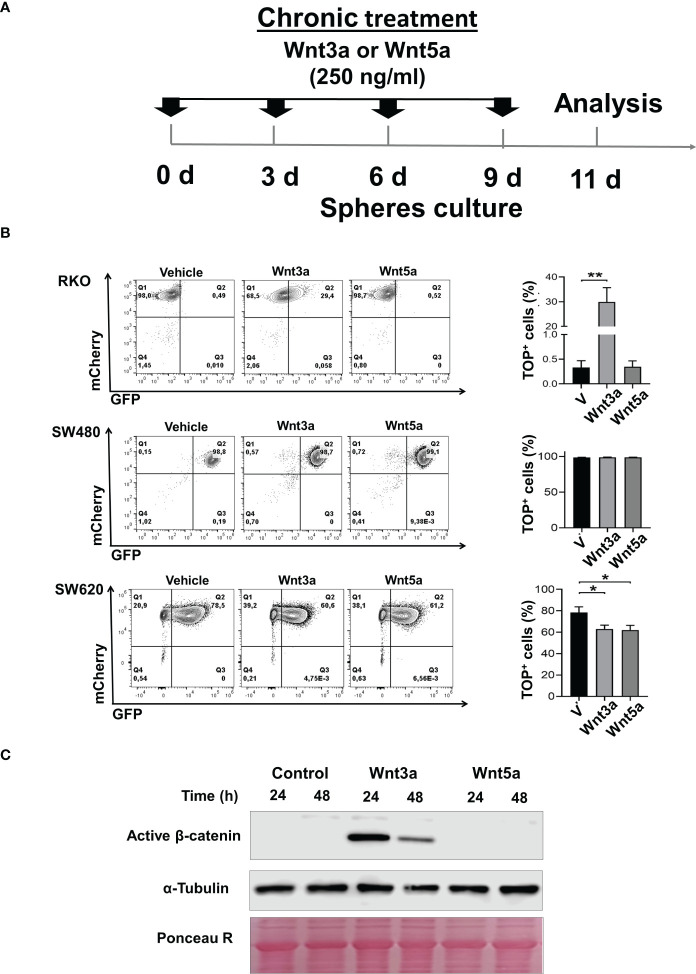
Wnt3a but not Wnt5a stimulates β-catenin transcriptional activity in RKO and SW620 sphere cells. **(A)**. Experimental scheme for spheres stimulation of RKO^TOP/GFP^, SW480^TOP/GFP^, and SW620^TOP/GFP^ cells with Wnt3a (250ng/ml) or Wnt5a (250ng/ml). Transduced cells are indicated as mCherry+ cells **(B)**. Density plots were measured by flow cytometry (left panel), and the quantification of the percentage of GFP^+^ cells of RKO^TOP/GFP^, SW480^TOP/GFP^, and SW620^TOP/GFP^ from spheres chronically treated with Vehicle, Wnt3a or Wnt5a are shown (right panel). **(C)** Analysis of active form of β-catenin by Western blot performed in samples obtained from RKO spheres (second generation) in the absence or the presence of Wnt3a or Wnt5a at the indicated times. α-Tubulin and Ponceau Red were used as loading controls. Data are represented as the mean values ± SEM of at least three independent experiments. Statistical analysis was performed using one-way ANOVA followed by Bonferroni´s multiple comparisons. *p<0.05; **p<0.005.

The response to Wnt ligands is partially dependent on mutations in components of the canonical Wnt pathway (16). In our work, we employed RKO and SW480 or SW620 human colon cancer cell lines as representative of normal Wnt signaling (RKO, which expresses normal APC protein) and constitutively active Wnt signaling (SW480 and SW620, which express a truncated version of APC and representative of distinct stage of progression in the same patient) (17). We have previously reported that in RKO cells, only prototype canonical Wnt3a is able to activate the β-catenin-mediated transcriptional activity in a dose-dependent manner and not Wnt5a, and this last one blocks the Wnt3a-induced β-catenin transcriptional activity (8). However, these studies were carried out in culture standard conditions of the monolayer. To examine if this response mediated by Wnt ligands is conserved in spheres, RKO, SW480, and SW620 sphere cells were transduced with pmCherry-TOP/GFP, which allows evaluating the β-catenin-dependent transcriptional activity by GFP expression. For stimulation, the sphere medium was supplemented with Wnt3a or Wnt5a and added each third day (**Figure 2A**). In agreement with data from monolayer conditions, results showed an increase in the percentage of GFP+ cells in RKO^TOP/GFP^ and also an increase in active β-catenin (non-phosphorylated) levels when second-generation spheres were stimulated with Wnt3a, but not with Wnt5a, then demonstrating responsiveness of canonical Wnt pathway by Wnt3a in RKO spheres (**Figures 2B, C**). In SW480^TOP/GFP^ spheres, as expected, there were no changes in the presence of Wnt3a or Wnt5a since these cells have ligand-independent, constitutive active canonical Wnt signaling. Furthermore, most SW480^TOP/GFP cells^ were GFP+, confirming that almost all of them have active canonical Wnt pathways (**Figure 2B**). SW620^TOP/GFP^ cells showed a different phenotype at basal conditions than RKO and SW480 because GFP+ and GFP- cells were clearly identified, indicating that, even though SW620 cells were derived from the same patient that SW480 cells, the control of canonical Wnt pathway is distinct: in the presence of Wnt3a or Wnt5a, the percentage of GFP+ cells diminished in a statistically significant manner, demonstrating that, indeed, the response of canonical Wnt signaling to Wnt ligands is different between SW480 and SW620 (**Figure 2B**). Thus, data indicate that the response to Wnt ligands is an intrinsic characteristic of the cells, and each cell line represents a unique context for regulating Wnt signaling.

### 3.3 Wnt3a or Wnt5a stimulate sphere formation in RKO and SW620 cells

**Figure 3:**
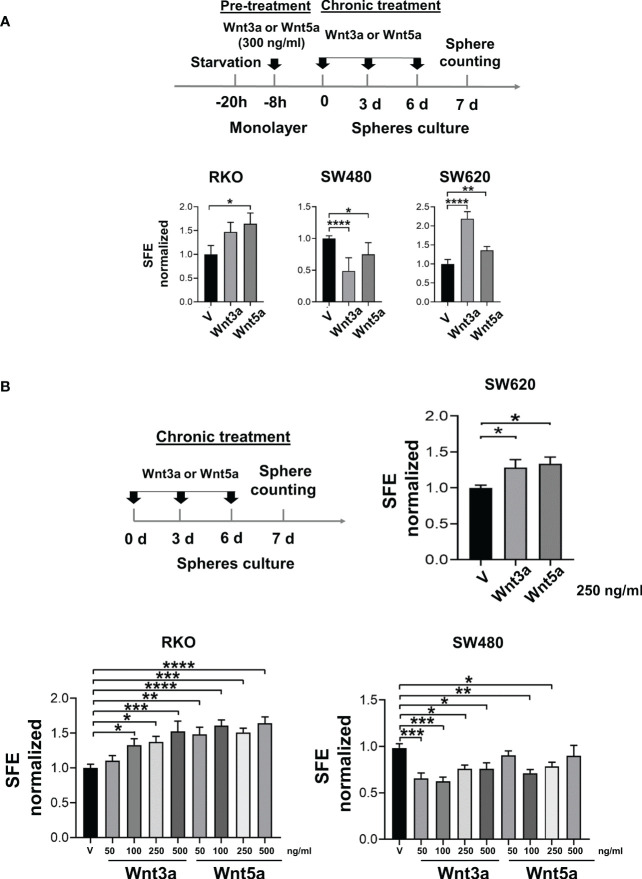
Wnt3a and Wnt5a stimulate spheres formation in RKO and SW620 but decrease in SW480. **(A)** Experimental scheme for spheres stimulation (upper panel) and quantification of SFE in RKO, SW480, and SW620 cells (lower panel) with pre-treatment and chronic treatment with Wnt3a or Wnt5a. **(B)** Experimental scheme for spheres stimulation (upper panel) and quantification of SFE in RKO, SW480, and SW620 cells (lower panel) with the chronic treatment of Wnt3a or Wnt5a at the indicated concentrations. Data are represented as the mean values ± SEM of at least three independent experiments. Statistical analysis was performed using one-way ANOVA followed by Bonferroni´s multiple comparisons. *p<0.05; **p<0.01; ***p<0.001; ****p<0.0001.

To know the effect of Wnt3a and Wnt5a in sphere formation, cells were pretreated with Wnt ligands in monolayer culture before seeding them to form spheres. Wnt ligands were also added every third day during the growth stage of spheres to maintain the stimulus. Our results showed that the SFE increased in both RKO and SW620 cells treated with Wnt3a or Wnt5a (**Figure 3A**). The positive effect of Wnt3a on sphere formation could be mediated in RKO cells due to the activation of the β-catenin transcriptional activity. However, the positive impact on SFE may not be mediated by a ligand-induced activation of β-catenin transcriptional activity since in both RKO and SW620 cells, Wnt5a also produced an increase in SFE (**Figure 3A**). In addition, SW620 also augmented SFE with Wnt3a or Wnt5a (**Figure 3A**), although β-catenin-mediated transcription was decreased in the presence of Wnt3a or Wnt5a in these cells (**Figure 2B**). Together, these results suggested an alternative β-catenin transcriptional activity-independent mechanism for inducing sphere formation capacity in RKO and SW620 cells in response to Wnt ligands (**Figure 3A**). In SW480 cells, a decrease in SFE was obtained upon Wnt3a or Wnt5a treatment (**Figure 3A**). In this case, since SW480 cells did not show changes in the canonical Wnt pathway in the presence of Wnt3a or Wnt5a, as observed in **Figure 2C**, the decrease observed in sphere formation induced by both Wnt3a or Wnt5a, again suggests that it may occur through a β-catenin-independent, but Wnt-dependent mechanism.

To confirm that these results were due to a direct effect of Wnt ligands in CSC-enriched cultures, the spheres were stimulated exclusively in the growth stage of spheres by chronic treatment. Results showed that both RKO and SW620 cells increased SFE when Wnt3a or Wnt5a were present (**Figure 3B**). In the case of RKO, the increase was dose-dependent, and although both Wnt ligands stimulated SFE, the changes induced by Wnt5a were more significant than the ones induced by Wnt3a, suggesting that each ligand may not necessarily activate the same signaling mechanisms. On the contrary, SW480 cells decreased SFE when the spheres were stimulated with Wnt3a or Wnt5a, being statistically significant in most of the concentrations evaluated (**Figure 3B**). These findings were consistent with the effects of Wnt ligands in pretreated spheres, and therefore, the impact of Wnt ligands on sphere formation cannot be explained by the levels of activation of the canonical Wnt pathway.

### 3.4 Wnt ligands affect the proliferative capacity of spheres in the short and long term

**Figure 4:**
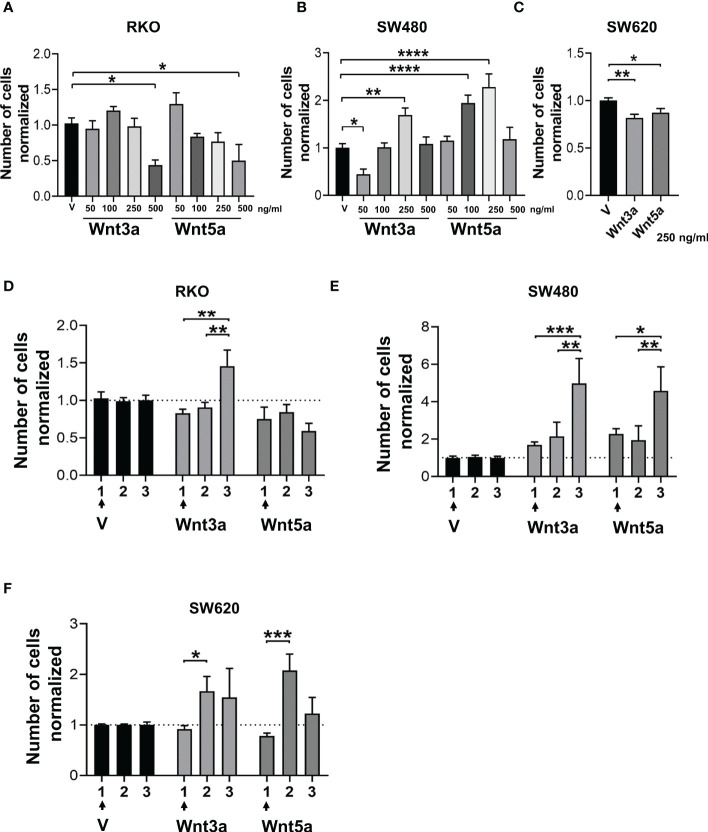
Effect of Wnt ligands on the proliferative capacity in spheres in the short and long term. **(A–C)** Normalized cell number obtained from the 1^st^ generation of spheres of RKO, SW480, and SW620 cells treated with Wnt3a or Wnt5a at the indicated concentrations. **(D–F)** Comparison of normalized cell number obtained from the first, second, and third generation of RKO, SW480, and SW620 spheres treated with Wnt3a or Wnt5a only in the first generation. Data are represented as the mean values ± SEM of at least three independent experiments. Statistical analysis was performed using one-way ANOVA followed by Bonferroni´s multiple comparisons. *p<0.05; **p<0.01; ***p<0.001; ****p<0.0001.

The proliferation in a serum-free medium, such as in sphere culture, is an important aspect of the study of CSC under these conditions. The cell number obtained from sphere culture in the absence or the presence of Wnt ligands was quantified to know whether Wnt ligands have an impact on this process. Results showed that at the highest concentration used of either Wnt3a or Wnt5a, the cell number was lower compared to the vehicle in RKO cells (**Figure 4A**), although an increase in SFE has been previously observed in results shown in **Figure 3B**, indicating, therefore, that forming spheres and cellular propagation are two processes that can be regulated differently by the same stimulus. This phenomenon was similar in SW620 cells, where the cell number decreased (**Figure 4C**) though their SFE increased (**Figure 3B**) under the same conditions of Wnt ligand stimulation. The idea of differential regulation between sphere formation capacity and proliferation is reinforced with the findings obtained in SW480 cells, where both ligands induced an increase in proliferation (**Figure 4B**), while SFE was reduced (**Figure 3B**).

Because Wnt ligands can evoke changes in cells that can be sustained over long periods (18), we were interested to know whether a chronic stimulation in the first generation of sphere formation might have an impact on subsequent generations, specifically on proliferation. The results showed that Wnt3a increased proliferation at the third generation in the three RKO, SW480, and SW620 spheres, although it was only statistically significant in RKO and SW480 spheres (**Figures 4D–F**). These data demonstrated that Wnt3a is closely related to positive regulation of cell proliferation, which was observed more clearly in subsequent generations than in the first generation, despite having stopped stimulating with Wnt ligands in the second and third generations. Strikingly, the fold change was greater in SW480 cells than in RKO and SW620 cells, even though SW480 cells did not display changes in the canonical Wnt pathway in the first generation, as shown in **Figure 2C**. On the other hand, Wnt5a maintained low levels of proliferation in the three generations of RKO cells while increasing in SW480 and SW620 in the third and second generations, respectively (**Figures 4D–F**). These findings highlight that the relationship between Wnt5a and proliferation depends on the cellular context. Notably, the fold change observed in SW480 cells was greater than in SW620 cells, corroborating that spheres of SW480 are responsive to both types of ligands, which has repercussions in the proliferative capacity.

### 3.5 The activity of PLC is essential for sphere capacity formation

**Figure 5:**
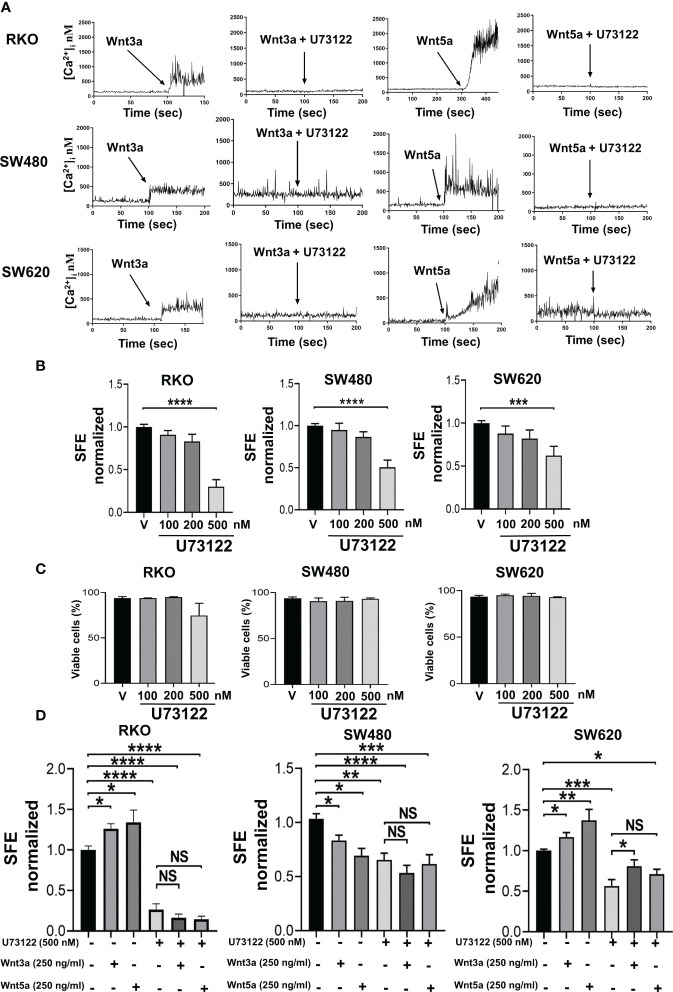
Inhibition of PLC impairs sphere formation. **(A)** Both Wnt5a or Wnt3a increase cytosol calcium concentration in RKO, SW480 or SW620 spheres that was blocked by pretreatment of sphere cells with PLC-specific inhibitor U-73122. Cells were serum-starved overnight and then loaded with 2.5 μM of Fura-2/AM in Krebs-Ringer-HEPES containing 0.05% BSA, pH 7.4, for 1 h at 37 ^a^C. Cells were washed and sphere cells stimulated with the conditioned medium containing Wnt3a or Wnt5a. Additionally, the spheres were pre-treated in the absence or presence of 4 µM PLC inhibitor U73122 before stimulation, as indicated. Traces are representative of three experiments using different cell preparations. **(B)** Quantifying SFE in spheres of RKO, SW480, and SW620 cells treated with the PLC inhibitor U73122 at the indicated concentrations each third day. **(C)** Quantification of viable cells of spheres of RKO, SW480, and SW620 under the same conditions as described in panel **(B)** Evaluation was performed by IP staining and detected by flow cytometry on the 11^th^ day of culture. **(D)** Quantifying SFE in spheres of RKO, SW480, and SW620 cells treated with U73122, Wnt3a, Wnt5a, or a combination of them. The concentration of each stimulus is indicated in the figure. Data are represented as the mean values ± SEM of at least three independent experiments. Statistical analysis was performed using one-way ANOVA followed by Bonferroni´s multiple comparisons. *p<0.05; **p<0.01; ***p<0.001; ****p<0.0001; NS, not significative.

As mentioned before, we recently reported that both canonical prototype Wnt3a ligand and noncanonical prototype Wnt5a ligand promote PLC-dependent Ca2+ mobilization and migration in both RKO or SW480 colon cancer cells under standard monolayer culture conditions (8). In this work, we confirmed that both Wnt3a and Wnt5a ligands promote Ca2+ mobilization that is blocked as a result of PLC inhibition in RKO, SW480, and SW620 spheres, as can be observed in **Figure 5A**. In addition, we found that these same ligands cause changes in the ability to form spheres. To know whether noncanonical Wnt/Ca2+ pathway is important in the regulation of sphere formation, the cells were treated with the PLC-specific inhibitor U73122 alone at different concentrations during sphere culture. Interestingly, the results showed that the percentage of SFE decreased in a dose-dependent manner at similar rates in all three cell lines tested (**Figure 5B**) despite the differences in the sphere formation capacity previously observed upon Wnt stimulation, suggesting that the role of PLC seems crucial for inducing and maintaining this capacity. The cell viability was measured to rule out if the effects of U73122 were due to toxicity mediated by the inhibitor. Quantification of living cells did not show statistically significant differences, even at the highest dose employed, demonstrating that PLC inhibition impairs CSC cultures without promoting cell death (**Figure 5C**). Because Wnt-dependent functions have been attributed to PLC, we next examine whether PLC inhibition could affect the changes in sphere formation induced by Wnt3a or Wnt5a. The spheres were then treated either with Wnt3a or Wnt5a along with 500 nM U73122. The results in **Figure 5D** show that U73122 decreased the SFE by a similar value indistinctly whether Wnt3a or Wnt5a was present or not (**Figure 5D**). In detail, the increase of SFE induced by Wnt ligands in RKO and SW620 cells was diminished, while in SW480 cells, in which the same stimulus decreased sphere formation, the levels of SFE remained low when U73122 was added in the absence or the presence of Wnt ligands (**Figure 5D**). These findings demonstrated that PLC activity is essential for sphere formation in these cell lines and that Wnt ligands could not compensate for the negative effect produced by PLC inhibition, suggesting that PLC is a downstream component in the signaling mediated by both ligands.

### 3.6 Sphere forming capacity also required the NFAT function

**Figure 6:**
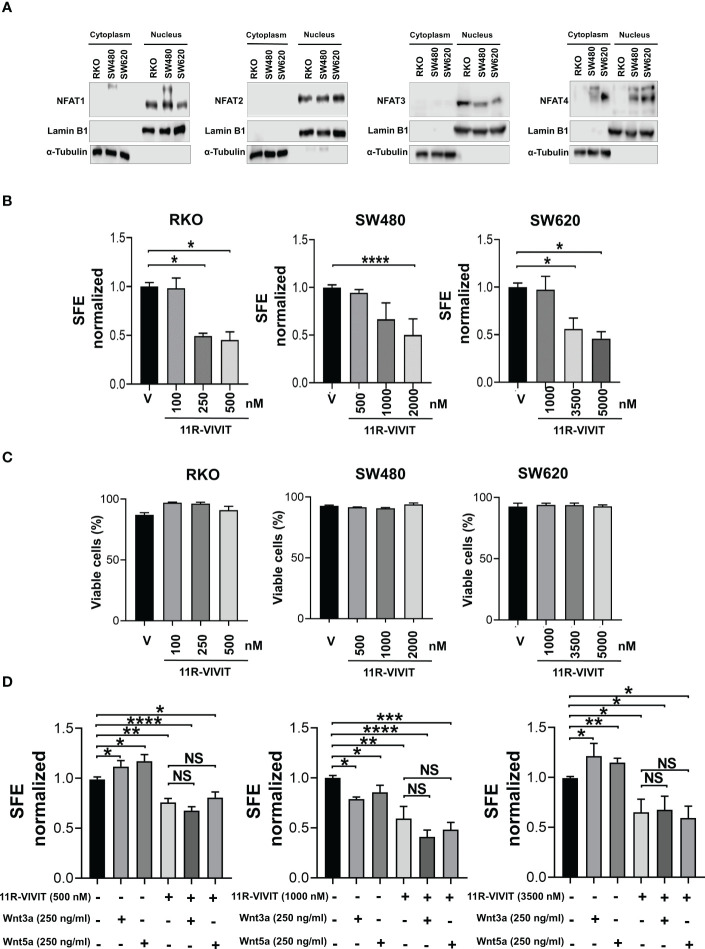
NFAT activity is required for spheres formation. **(A)** Analysis of NFAT1, NFAT2, NFAT3, and NFAT4 expression by Western blot performed in samples obtained from cell fractionation from monolayer cultures. Lamin B1 and α-tubulin were used as a loading control and fractionation purity of nuclear and cytoplasmic fractions, respectively. **(B)** Quantifying SFE in spheres of RKO, SW480, and SW620 cells treated with the NFAT inhibitor 11R-VIVIT at the indicated concentrations each third day. **(C)** Quantification of viable cells of spheres of RKO, SW480, and SW620 under the same conditions described in panel **(B)** Evaluation was performed by IP staining and detected by flow cytometry on the 11^th^ day of culture. **(D)** Quantifying SFE in spheres of RKO, SW480, and SW620 cells treated with 11R-VIVIT, Wnt3a, Wnt5a, or their combination. The concentration of each stimulus is indicated in the figure. Data are represented as the mean values ± SEM of at least three independent experiments. Statistical analysis was performed using one-way ANOVA followed by Bonferroni´s multiple comparisons. *p<0.05; **p<0.01; ***p<0.001; ****p<0.0001; NS, not significative.

The noncanonical Wnt/Ca2+ pathway has implications in transcriptional activation through NFAT family members. This family of transcription factors consists of NFAT1 (NFATc2), NFAT2 (NFATc1), NFAT3 (NFATc4), NFAT4 (NFATc3), and NFAT5. Therefore, we first elucidate the expression profile of NFATs in colon tumor spheres, in addition to knowing their activation by distinguishing their location at the nucleus. The subcellular fractions of spheres did not allow the detection of NFATs, possibly due to the low amount of protein, so the evaluation was performed in subcellular fractions of monolayer cultures. Results demonstrated that NFAT1, 2, 3, and 4 are expressed in RKO, SW480, and SW620 cells (**Figure 6A**). Remarkably, NFATs were found enriched in the nucleus, suggesting they have constitutive activity in basal (unstimulated) conditions.

Subsequently, the activity of NFATs was blocked using 11R-VIVIT, a cell-permeable NFAT inhibitor (suitable for inhibiting all NFAT isoforms), during the sphere growth stage, and the SFE was measured. The capacity to form spheres in RKO, SW480 and SW620 cells was blocked with 11R-VIVIT in a dose-dependent manner. Notably, each cell line required a different concentration of 11R-VIVIT to induce the loss of 50% in SFE, being SW620 cells which required the highest concentration of 11R-VIVIT, followed by SW480 cells, and RKO cells required the less concentration, which suggests that the sensitivity to 11R-VIVIT is possibly due to the characteristics of each cell line (**Figure 6B**).

Again, to investigate if 11R-VIVIT was affecting the viability of the cells, which could explain the decrease in the sphere-forming capacity, we measured dead cells by IP staining. As shown in **Figure 6C**, flow cytometry analysis demonstrated similar percentages of cell survival in each concentration of compound employed, thus indicating that the outcome of 11R-VIVIT was not due to toxicity (**Figure 6C**). Since it was established that 11R-VIVIT reduced sphere formation, we examined the SFE in co-treatment of 11R-VIVIT with Wnt3a or Wnt5 to know if they could induce any compensatory effect. We found that the exposure to 11R-VIVIT (IC_50_ dose for each cell line) decreased the SFE, regardless of the presence of either Wnt ligand, showing that NFAT activity is also crucial for the sphere-forming capacity. In addition, these results also indicate that Wnt3a or Wnt5a stimulate a noncanonical Wnt-Ca2+ pathway since NFAT acts downstream of Wnt3a or Wnt5a stimulation of spheres (**Figure 6D**).

## 4 Discussion

Cancer stem cells (CSCs) or tumor-initiating cells have been the focus of cancer research during the last years because they have been regarded as the cells of origin of cancer and are crucially involved in metastatic dissemination, resistance to cancer therapy, and disease recurrence (2–4). CSCs possess abilities generally associated with embryonic or adult stem cells, especially self-renewal and differentiation. In this work, we have used sphere culture, a widely used method to evaluate the self-renewal capacity of CSC. Despite the fact that the crucial role of the canonical Wnt pathway in self-renewal has been consistently reinforced, little is known about the involvement of β-catenin-independent Wnt pathways in this process. Our results demonstrate here that the non-canonical Wnt/Ca2+ cascade is essential for CSC self-renewal, regardless of the activation status of the canonical Wnt pathway. Furthermore, the cell proliferation was stimulated by Wnt3a and Wnt5a without necessarily activating β-catenin-mediated transcription, which indicates that CSC requires non-canonical Wnt pathways.

CRC is considered an aberrant Wnt pathway disease because APC mutations are the earliest alteration detected in most colon cancer cases (3, 4). Considering this, we have used in our studies SW480 cells and its metastatic derivative SW620 cell line for being cancer cells with truncated APC representing distinct stages of progression in the same patient. We also employed RKO cells, as it harbors wild-type APC, and thus an inducible activation of the canonical Wnt pathway. This allows us to broaden the outlook of the implications of non-canonical Wnt pathways in different cellular contexts.

We found here that SW480 spheres no longer increased β-catenin-mediated transcription in the presence of Wnt3a, but this same ligand markedly promoted proliferation, suggesting the existence in these cells of Wnt-responsive non-canonical pathways. Remarkably, both Wnt3a and Wnt5a were capable of stimulating cell proliferation. The effects of Wnt ligands on proliferative capacity were evidently distinguishable in later generations of spheres, corroborating that Wnt ligands can have long-time outcomes. Several reports have exemplified that functional changes can be found by the passing of spheres (12, 15). For example, stimulation of hematopoietic stem cells (HSC) with Wnt5a during six days favored the short-term and long-term repopulating ability of HSC over 16 weeks from the latter, thus indicating that a Wnt stimulus can dispose cells to lasting changes (15). SW620 is a cell line with mutated APC since it comes from the same patient as SW480 but was isolated from a lymph node due to metastasis. Differences between SW480 and SW620 have been recognized here, which implies that they do not share identical biological characteristics (16).

The most salient finding obtained in this work was the demonstration that Wnt3a and Wnt5a ligands stimulate the non-canonical Wnt/Ca2+ pathway activating PLC and NFAT transcription factors to critically regulate the self-renewal capacity of CSCs. In this regard, the role of non-canonical Wnt signaling in CSCs was reported before by Qin L et al. in 2015 (17). They found that Wnt5a promoted epithelial-mesenchymal transition (EMT) in nasopharyngeal carcinoma (NPC) cells and induced the accumulation of CD24-CD44+ cells and side population, suggesting that Wnt5a is an important molecule in promoting stem cell characteristics in this cancer type (17). In addition, Yang J et al., in 2016 (18) showed that Wnt5a could increase the aldehyde dehydrogenase (ALDH) positive lung cancer stem cells inducing an enhanced capacity of cell proliferation, migration, invasion, and colony formation.

Based on our results, despite the contrasted alterations of sphere formation and proliferation in response to Wnt ligands between cell lines, the role of Wnt/Ca2` turned out to be fundamental in CSC of CRC. The calcium signaling downstream of PLC has also been established as relevant for features of embryonic stem cells (ESC) such as self-renewal and pluripotency, although upstream components have remained poorly understood. At the membrane level, mGlu (metabotropic glutamate) receptors increased intracellular calcium in mESC, and dietary L-glutamate encouraged the proliferation of intestinal stem cells (ISC) in Drosophila, indicating mGlu receptor-dependent calcium oscillations (19). On the other hand, LPA (Lysophosphatidic acid) has been reported to increase calcium levels in mESC, augmenting the proliferation of ESC. In this study, the PLC inhibitor U73122 blocked the effect of LPA, implicating PLC activity. Noteworthy, the effect of U73122 on the proliferation of ESC was not due to the toxicity of the compound (20, 21), similar to our data, in which U73122 decreased the sphere formation efficiency without affecting cellular viability. Activation of G-protein coupled receptors (GPCR) by culture media components has been proposed to be part of the signaling pathway in ESC, in which fluctuations of calcium levels are described (21). Specifically, G(α-q/11)-coupled GPCR can compensate for the absence of bFGF for hESC self-renewal, in which PLCβ and CAMKII are downstream components (22).

Regarding what is known about the role played by the non-canonical Wnt/Ca2+ pathway in colon cancer patients, accumulating evidence has shown that the prototypal non-canonical Wnt5a ligand-mediated actions, along with an increase in Ror1/Ror2 non-canonical co-receptors expression have been found to be associated with an aggressive phenotype in colon cancer patients. However, specifically, the role(s) of the Wnt/Ca2+ pathway has not been studied so far in patients. Nevertheless and interestingly, using the American Joint Committee on Cancer, TCGA public data from Pan-Cancer, and the Gene Expression Profiling Interactive Analysis (GEPIA2) platform, we performed an *in silico* analysis of some critical Wnt/Ca2+ components. The analysis of CAMK2B mRNA in different stages of colon cancer patients and the analysis of Kaplan-Meier curves of survival corresponding to NFAT1, NFAT2, NFAT3, and NFAT4 expression signatures demonstrated that all these Wnt/Ca2+ elements are overexpressed in advanced colorectal cancer stages, inducing a reduced survival time in patients (please see **Supplementary Figure 3**).

A more direct relationship of intracellular calcium oscillations with properties in CSC has begun to emerge, highlighting that PLC is crucial in the stemness of both normal and cancer cells. Interestingly, in 2015 Cecchetti S et al. (23) reported that the exposure of tumor-initiating cells in squamous cell carcinoma to the phosphatidylcholine-specific Phospholipase C inhibitor D609 interfered with the proliferation and survival of these cells (23). But a most direct implication of PLC activation in stimulating embryonic stem cell proliferation, although by an unknown ligand, was reported in 2003 by Quinlan RL et al. (24), who consistent with our findings, reported that the PLC inhibitor U-73122 significantly reduced the number of mouse ES cells in a dose-dependent manner but did not decrease cell viability or increase the incidence of apoptotic cells, indicating that PLC has a controlling role in ES-cell proliferation (24). It is noteworthy that this experimental evidence, along with that presented here, suggests that the Phosphatidylinositol (PtdIns) signaling system is essential for the normal proliferation of ES cells and for regulating self-renewal capacity in CSCs. Interestingly, it has been reported very recently that recruitment of Dvl to the plasma membrane can increase the local PI (4,5)P2 concentration, which is the PLC substrate (25). These authors suggest a positive feedback loop in which Wnt-stimulated local PI (4,5)P2 production enhances Dvl recruitment and further PI (4,5)P2 production to support Dvl polymerization and Wnt-mediated signaling.

Altogether, our results demonstrate that Wnt ligands can improve characteristics of CSC of CRC in a β-catenin-independent manner, supporting that non-canonical Wnt pathways are essential in these types of cells. In detail, we have characterized a Wnt/Ca2+ PLC/NFAT signaling pathway as a key regulator pathway for the biological properties of CSC, which expands the panorama of the complex Wnt network beyond the canonical pathway.

## Data availability statement

The original contributions presented in the study are included in the article/**Supplementary Material**. Further inquiries can be directed to the corresponding author.

## Author contributions

MS-S and MR-F participated in the experimental design and writing. MS-S, AM-L, MC-P, EA-O, and JM-M performed the experiments. MS-S, AM-L, and MR-F analyzed the data. MR-F provided the financial support. MR-F supervised and approved the final version of this manuscript. All authors contributed to the article and approved the submitted version.

## Supplementary material

Supplementary Figure 1 CSC-related markers are expressed in spheres cultures. **(A)** Density plots showing the proportion of CD133, CD44 and CD44v6 in spheres of RKO, SW480 and SW620 cells were measured by flow cytometry.

Supplementary Figure 2 Cell viability is not affected by U73122 or 11R-VIVIT. Density plots of viable cells of spheres of RKO, SW480, and SW620 cells treated with U73122 **(A)** or 11R-VIVIT **(B)** at the indicated concentrations each third day, evaluated by IP staining and detected by flow cytometry on the 11^th^ day of culture.

Supplementary Figure 3 Components of Non-canonical Wnt pathway are associated with clinical parameters. Kaplan-Meier curves of survival for Signature: *NFAT1*, *NFAT2*, *NFAT3* and *NFAT4* **(A)** and *CAMK2B* **(B)** in patients with CRC are shown. **(C)** Analysis of CAMK2B mRNA in different stages of CRC according to American Joint Committee on Cancer. Data are represented as Z-scores relative to all samples. Statistical analysis was performed using Mann-Whitney U test non-parametric. *p<0.05; **p=0.0015; ****p<0.0001
