# Supplementary figures and images for: Non-canonical Wnt/Ca2+ signaling is essential to promote self-renewal and proliferation in colon cancer stem cells
Source: Front Oncol. 2023 Mar 10;13:1121787. doi: 10.3389/fonc.2023.1121787 (PMC10036746; doi:10.3389/fonc.2023.1121787)

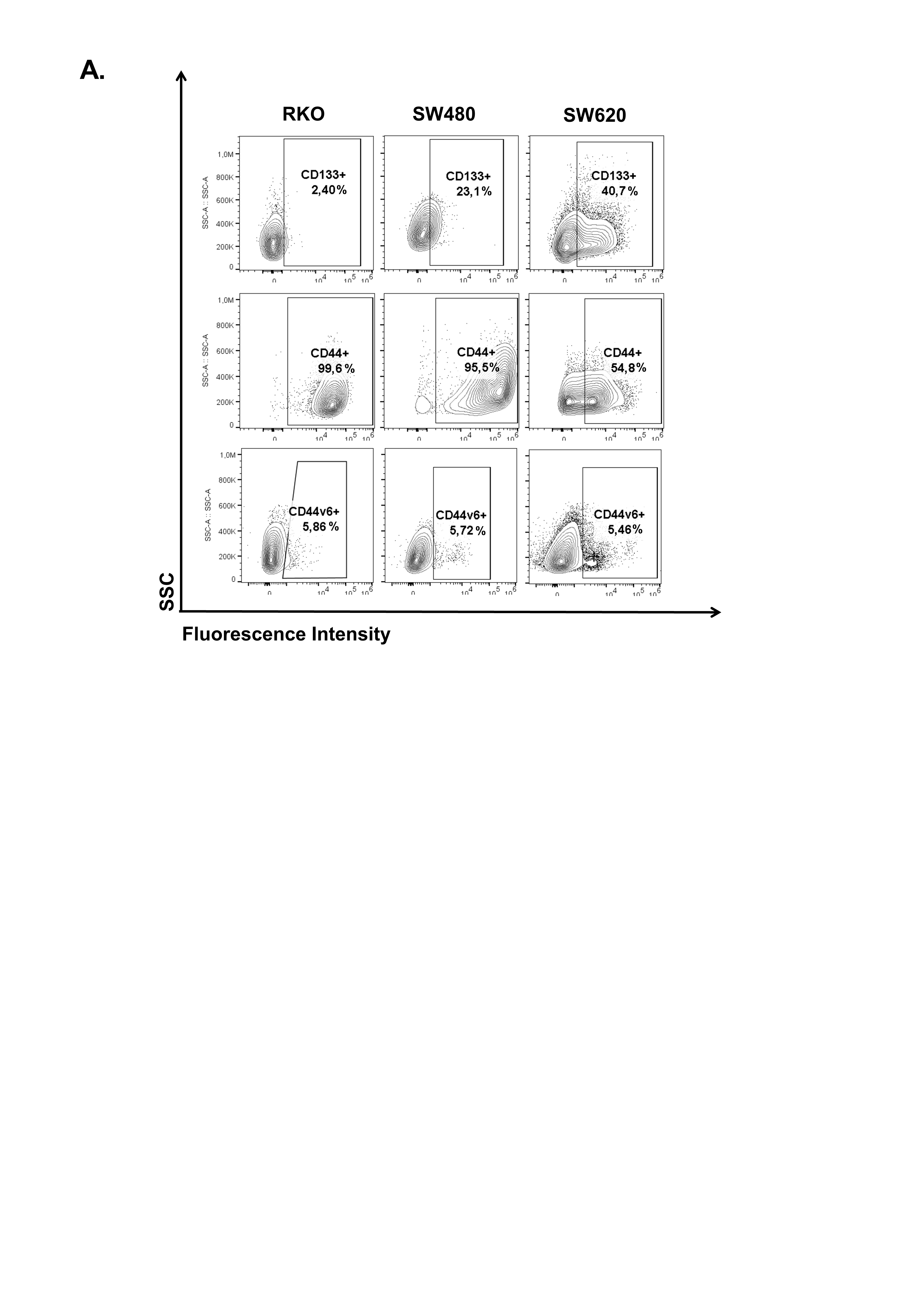

Supplement: Supplementary Figure 1 — CSC-related markers are expressed in spheres cultures. (A) Density plots showing the proportion of CD133, CD44 and CD44v6 in spheres of RKO, SW480 and SW620 cells were measured by flow cytometry. [file Image_1.tif]

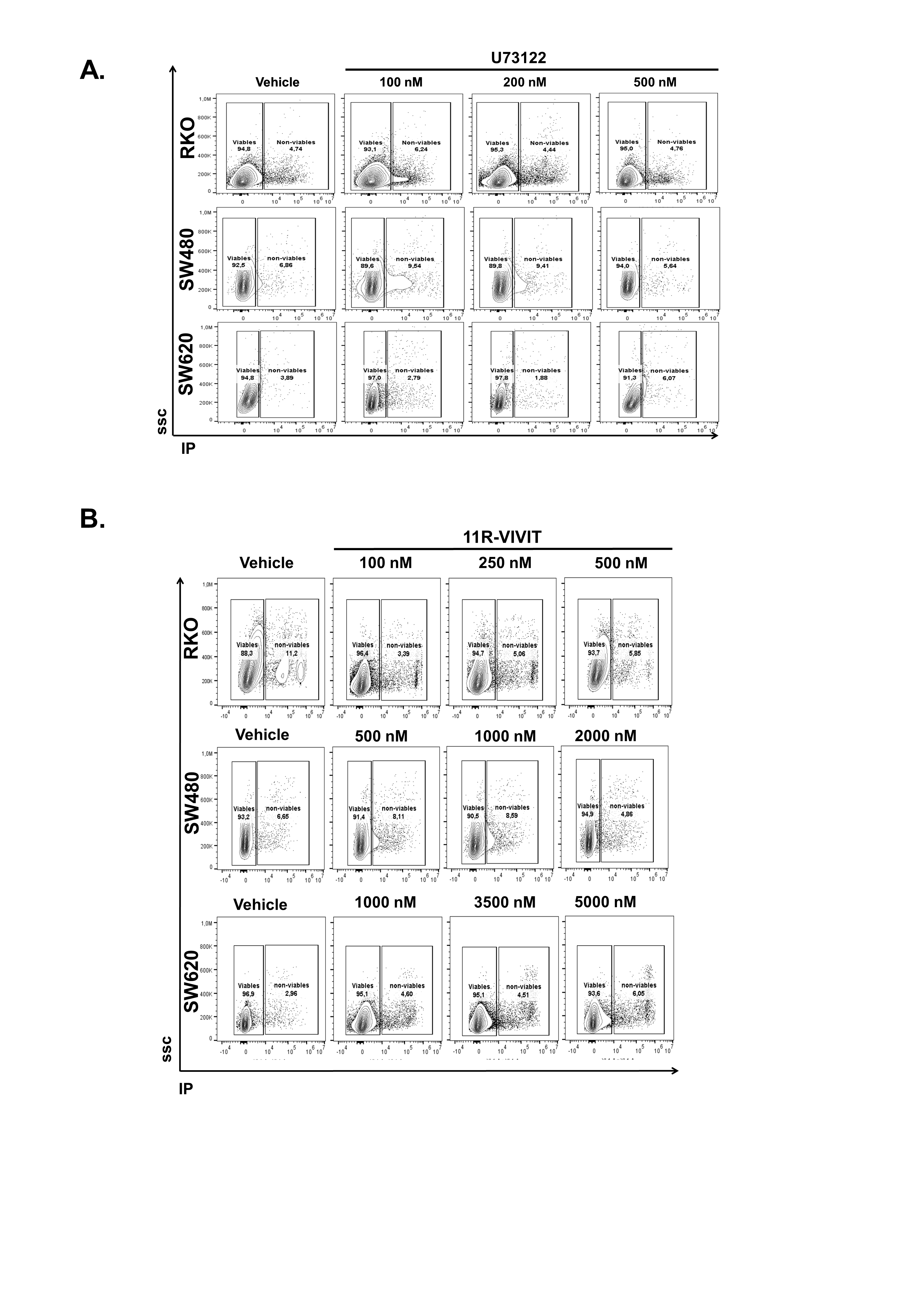

Supplement: Supplementary Figure 2 — Cell viability is not affected by U73122 or 11R-VIVIT. Density plots of viable cells of spheres of RKO, SW480, and SW620 cells treated with U73122 (A) or 11R-VIVIT (B) at the indicated concentrations each third day, evaluated by IP staining and detected by flow cytometry on the 11th day of culture. [file Image_2.tif]

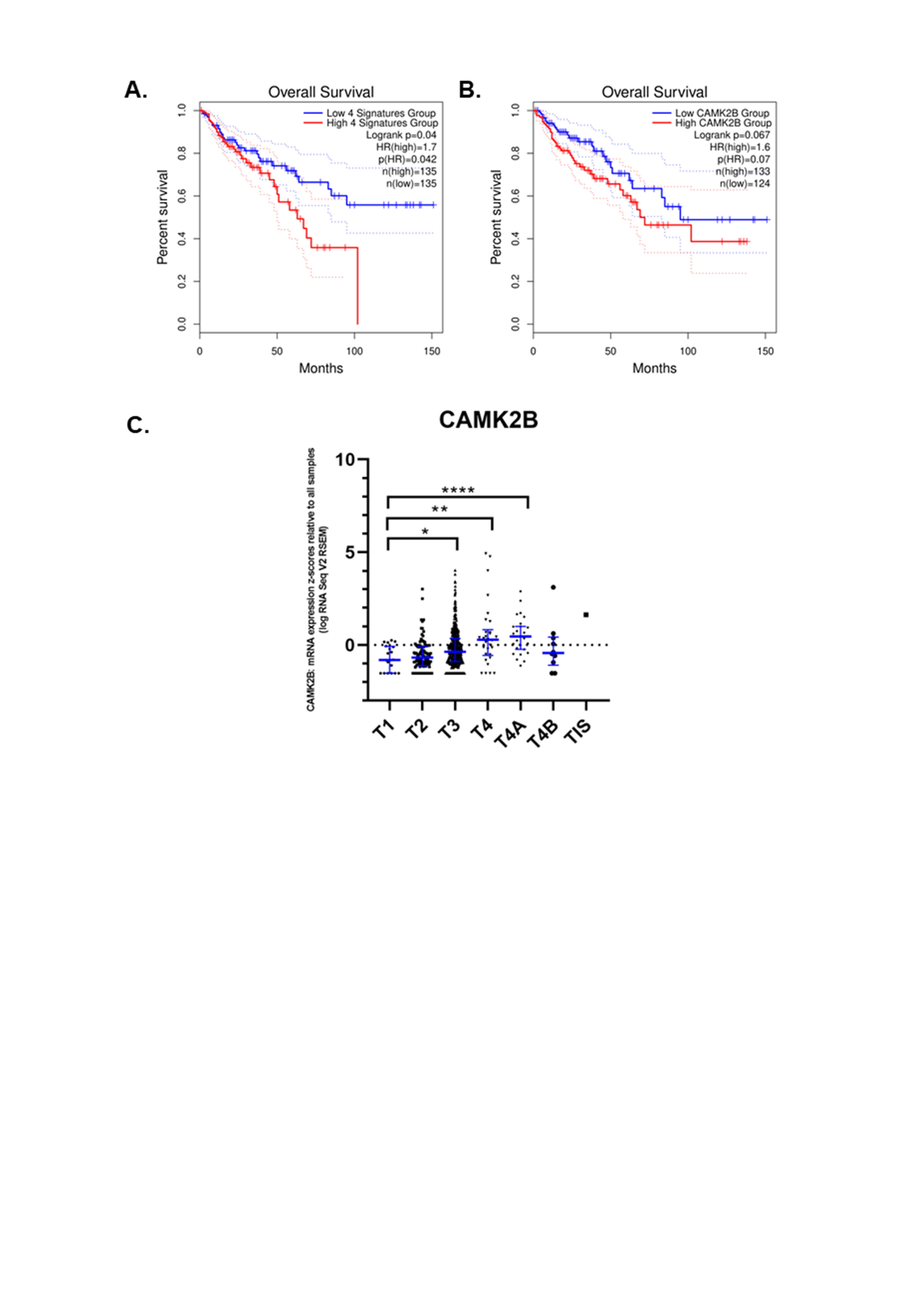

Supplement: Supplementary Figure 3 — Components of Non-canonical Wnt pathway are associated with clinical parameters. Kaplan-Meier curves of survival for Signature: NFAT1, NFAT2, NFAT3 and NFAT4 (A) and CAMK2B (B) in patients with CRC are shown. (C) Analysis of CAMK2B mRNA in different stages of CRC according to American Joint Committee on Cancer. Data are represented as Z-scores relative to all samples. Statistical analysis was performed using Mann-Whitney U test non-parametric. *p<0.05; **p=0.0015; ****p<0.0001 [file Image_3.tif]
